# Counter-Irritation as a Means to Co-Ordination

**Published:** 1921-05-21

**Authors:** 


### Counter-Irritation as a Means to Co-ordination.

### DR. MACNAMARA'S METHOD.

BY A HOSPITAL MATRON.

It would almost seem that when Dr. Macnamara, the Minister for Labour, ruled "that nurses in voluntary hospitals are employed persons within the meaning of the Unemployment Insurance Act, 1920," he wished to impress upon members of the Nursing Services the value of counter-irritation as a means to relieving deep-seated congestion. It is a curious fact that women who from the very commencement of their training are taught to observe, to annotate, and to act swiftly during an emergency, should utterly fail to apply these fundamental principles of their calling to the internal management of their business affairs. Instead of fostering in the minds of their pupils a strong professional spirit and a high sense of duty to their fellow-nurses, matrons and sisters have preferred to inculcate a sense of self-depreciation and a lack of responsibility towards the problems of the present day. They have failed to make it clear that individual opinion, concisely expressed, is essential to the well-being of each section of the community, and is the first step towards the alleviation of outstanding abuses. The science of nursing can never develop in the highest and the widest sense until nurses realise the financial value of their work.

This is the lesson Dr. Macnamara has taught us. By including nurses in the Unemployment Insurance Act he has read a parable and pointed a moral; it is for the nursing services to profit by the rebuke they have received. After years of striving, nurses won the right to State Registration—an important step towards the official recognition of their professional status. It was accepted as evidence of a desire on the part of the Government to raise the educational standard of nursing and to assist the matrons of hospitals to obtain suitable candidates for training as probationary nurses; but the Minister for Labour, by ruling "that nurses are employed persons within the meaning of the Unemployment Insurance Act, 1920," has excluded nursing from amongst *the professions* now open to women, and has thus gone far to render null and void the Nurses' Registration Act, 1919, before the register was even opened. Fortunately his ruling has roused the individual nurse to a sense of wider responsibility. The reaction against self-depreciation has already set in; the perception that professional salvation can be found only in co-ordination is rapidly spreading.

The effect of Dr. Macnamara's ruling on the future of the nursing services is likely to be deep and lasting. The memory of an injustice rankles; it acts as a spur, and may prove a decisive argument in favour of amalgamation. It is for the College of Nursing to seize the present opportunity. The seeds of a profitable harvest have been sown broadcast throughout the land; the time to garner the corn has arrived. The local centres must be prepared to accept their responsibilities and to inaugurate a campaign of propaganda in the surrounding districts. Properly handled, this should prove the first step towards devolution and democratic government in the nursing profession—a milestone on the road to professional unity.

